# Molecular biology of breast tumors and prognosis

**DOI:** 10.12688/f1000research.8158.1

**Published:** 2016-04-21

**Authors:** Gustavo Baldassarre, Barbara Belletti

**Affiliations:** 1 Department of Experimental Oncology, Department of Translational Research, CRO Aviano IRCCS, National Cancer Institute, Italy, Aviano, Italy

**Keywords:** breast cancer, breast cancer treatment and care, breast cancer prognosis

## Abstract

Breast cancer is the most common cancer among women worldwide. Great scientific, economical, and organizational efforts are in place to understand the causes of onset, identify the critical molecular players of progression, and define new lines of intervention providing more benefits and less toxicity. These efforts have certainly not been vain, since overall survival, especially in specific subsets of breast cancer, has greatly improved during the last decades. At present, breast cancer patients’ treatment and care have reached a high standard of quality, and currently one of the most urgent needs resides in the necessity to better distinguish the tumors that need to be more aggressively treated and identify the best therapeutic option tailored to each patient. This objective will be achievable only if the information clarifying the biology of breast cancer can be successfully transferred to the clinic. A common effort by scientists and clinicians toward this integration and toward the use of multidisciplinary approaches will be necessary to reach this important goal.

## Introduction

Breast cancer (BC) is the most common cancer among women worldwide, accounting for approximately one-quarter of all cancers in females worldwide and 27% of cancers in developed countries with a Western lifestyle, thus representing a real health emergency ^1^. Here, we briefly focus on some specific biological and clinical aspects of BC that are still matter of controversy and in which there is urgent need for competent integration to implement diagnostic and therapeutic options.

## Gene expression profiles define different breast cancer subtypes

Large-scale gene expression profile (GEP) studies demonstrated that BCs are not a single entity but can be divided into at least four major subtypes: luminal A (LBC-A), luminal B (LBC-B), HER2-positive, and triple negative/basal-like ^2^. This classification has been recently confirmed and integrated with genomic data demonstrating that the diverse subtypes are indeed associated with different recurrent genomic alterations ^3^. LBC in the Western world represents the most common subtype, accounting for more than 60% of all diagnosed BC. In clinical practice, a few characteristics, such as estrogen receptor (ER), progesterone receptor (PR), HER2, and Ki67 expression, are currently used to distinguish LBC-A (ER+ and/or PR+, HER2-, low Ki67) from LBC-B (ER+ and/or PR+, HER2- or HER2+, high Ki67) ^4^. This classification has therapeutic implications, since based on the relative expression of the above markers, LBC patients will or will not receive hormone-, chemo-, or targeted-therapies ^4^. When compared with LBC-A, LBC-B displays a higher rate of early recurrence and worse prognosis, representing a subgroup for which the choice of the optimal therapy still represents a difficult task for the clinician ^4^. In fact, clear biomarkers to select the most appropriate (hormone- with or without chemo-) therapy in LBC-B still have to be validated and introduced into the clinic ^4^.

The above-mentioned molecular classification also has prognostic relevance, with triple-negative and HER2+ BC having a more aggressive progression. Moreover, since the same molecular characteristics are already present in the first stages of BC and in *in situ* lesions, it is expected that early diagnosis could help in the identification (and removal) of potentially malignant tumors that need to be aggressively treated.

## Significance of identifying *in situ* breast cancer and the risk of overtreatment

Based on this idea, several BC screening programs have been introduced worldwide in women aged >50 years. The introduction of screening programs has substantially increased the number of early stage *in situ* lesions, mainly ductal carcinoma *in situ* (DCIS). It is debated whether low-grade DCIS would result in an invasive stage ^5^. The general view was that the increase in DCIS diagnoses would result in decreased incidence of invasive BC and, eventually, decreased mortality for BC. However, this has not always been the case, as demonstrated by epidemiological analyses, raising some concern over the possibility of “over-diagnosis” linked to the wide spread of BC screening programs ^6^.

The importance of early diagnosis in BC was recently addressed by a large observational study estimating the mortality for BC following a diagnosis of DCIS ^7^. This important study, enrolling more than 100,000 women, provided the interesting observation that women diagnosed with DCIS display a BC-related death risk at 20 years comparable to that estimated for the general population and that using aggressive treatments for all DCIS does not reduce the mortality for BC ^7^. Yet a diagnosis of DCIS in black women or in women under the age of 40 and the presence of high risk factors such as HER2 expression are associated with an increased risk of BC-related death ^7^. This clinical evidence reinforces the concept that, even when *in situ*, different molecular alterations in BC strongly impact on the outcome of the disease and on patients’ survival.

## Breast cancer: age matters

This concept introduces the relevance of molecular studies to precisely identify the cancer that needs to be aggressively treated and to discover the most appropriate treatment for each patient. Addressing these two unmet clinical needs is the only way to further improve BC cure while limiting the risk of overtreatment.

The recent study by Narod and colleagues suggests that the group of BC patients that receives a diagnosis of BC before the age of 40 is the one that more urgently needs to be accurately classified at molecular level ^7^. Young age at diagnosis has emerged as an independent factor associated with higher risk of relapse and death in several large studies on BC ^8, 9^. Several factors have been linked to this poor prognosis, including large tumor size at diagnosis, higher tumor grade, mitotic index, lymphovascular invasion, increased expression of HER2, and lower ER and PR expression ^10^. More recently, it has been proposed that for these patients it is also worth testing the presence of mutation in the BC susceptibility genes *BRCA1* and *BRCA2*, independently of their family history ^11^. A general consensus in the scientific community has been reached to define BC in Young Women (BCYW), although fairly rare (~7% of all diagnosed BC), as a distinct entity that merits being studied and treated using specific guidelines and following specific research priorities ^12^.

More aggressive subtypes are more common in BCYW. In particular, when compared with older patients, BCYW more frequently displays the triple-negative and HER2+ BC subtypes. However, in the Western world, LBC-B still accounts for more than 60% of all BCYW, remaining the most common histotype and displaying a particularly bad prognosis ^8, 9^. These observations raised the question of whether BCYW has a unique biology or whether this just represents a surrogate of the higher incidence of aggressive molecular subtypes. But, even after correction for stage and tumor characteristics, young age at diagnosis remains an independent risk factor for relapse and BC-related death ^13^.

Unraveling the biological uniqueness of BCYW is fundamental, as it not only increases our understanding of the disease process but also underlies the decision of whether or not to offer the same therapeutic options reserved to the high-risk older patients or to choose therapeutic approaches based on a specific biology ^14^.

Accumulating evidence suggests that differences in the mammary stroma composition and changes that occur with pregnancy and breastfeeding likely contribute to the different biology of BCYW. Moreover, these tumors are enriched with processes related to immune-related gene signatures and immature mammary cell populations (RANKL, c-kit, BRCA1-mutated phenotype, mammary stem cells, and luminal progenitors) ^15^.

The comprehensive analysis of BC with respect to age on a large compendium of publicly available gene expression datasets (more than 3500 BCYW) ^15^ has demonstrated that specific pathways are altered in BCYW with respect to older patients. However, the identification of altered signaling pathways is not sufficient to identify the driver alterations responsible for tumor onset and/or progression.

A recent conference centered on the management of BCYW confirmed that, although some progress has been made in the understanding of the clinical and biological behaviors of BC in patients younger than 40, we still need to clarify many aspects to properly treat this particular subgroup of patients ^16^. In particular, there is a consensus on the fact that biology appears to be different in BCYW and that this is particularly true for the endocrine-sensitive tumors. Based on this consideration, it is necessary to study the tumor genetic profile in larger cohorts to further understand if a common and unique pattern of gene expression exists in BCYW. Then, it becomes clear that the study of primary tumors will not be sufficient and that characterizing the recurrent tumors will be mandatory. Finally, it could be of special relevance to understand the interaction of the endocrine and immune systems in these young patients and to cleverly look at response patterns to the current treatments. Only in this way will we have the possibility to improve the treatment, quality of life, and survival of BCYW patients ^16^.

## Is it the time, in the post-genomic era, to return to functional studies?

It is now clear that sequencing and gene expression profile studies must be associated with high-throughput functional analyses to precisely identify the genes and/or the pathway each tumor type is addicted to. A recent resource has proposed that it is possible to identify the vulnerability of each type of BC using functional assays, even using a panel of cell lines, if these well recapitulate the original disease ^17^.

We are convinced, however, that these types of assays should be integrated by the generation of more reliable models of validation. We recently explored this possibility focusing on the onset of local recurrences in BC by setting up a mouse model closely resembling the course of the human pathology ^18^. Our work highlighted that targeting specific signaling pathways at the time of surgery has great potential to prevent the re-appearance of BC in mice ^18– 21^. In particular, we observed that the specific inhibition of p70S6K1 had little effect on blocking the growth of established breast tumors but prevented the onset of BC recurrences when therapy was administered with a perisurgical treatment schedule. p70S6K1 activity was necessary for the survival of isolated BC cells residually present in the post-surgery setting, making it an ideal target to improve the efficacy of surgery ^18, 19^. Similar results were also shown for the PAR-4 protein using different models of recurrence formation ^22^. In these models, PAR-4 acted as an inhibitor of recurrence formation by inducing multinucleation in oncogene-addicted cells ^22^.

In accord with the importance of timely delivery of the therapy, it has been recently demonstrated that the application of intraoperative radiotherapy (IORT) in BC patients has different effects if used immediately after tumor removal or as a second procedure after pathological examination ^23^. At the molecular level, this clinical observation could be explained by the recently demonstrated direct effect of IORT in the tumor microenvironment, where it modulates the EGF-EGFR-p70S6K1 signaling axis, via the induction of miR-223 expression in the local peri-tumoral microenvironment ^21^.

It would be interesting to evaluate whether in high-risk BC patients, such as BCYW, similar mechanisms of cell survival exist and are at least partially responsible for their aggressive phenotype and whether targeting these specific pathways at the right time could significantly impact on the patient’s disease-free and overall survival. To this aim, better models recapitulating the biology of BCYW and more integration between clinicians and preclinical researchers are primarily and urgently needed.
